# Modeling Intermolecular Coulombic Decay with Non-Hermitian Real-Time Time-Dependent Density Functional Theory

**DOI:** 10.1021/acs.jpclett.4c01146

**Published:** 2024-07-25

**Authors:** Yi-Siang Wang, James X. Zhong Manis, Matthew C. Rohan, Thomas M. Orlando, Joshua S. Kretchmer

**Affiliations:** School of Chemistry and Biochemistry, Georgia Institute of Technology, Atlanta, Georgia 30332, United States

## Abstract

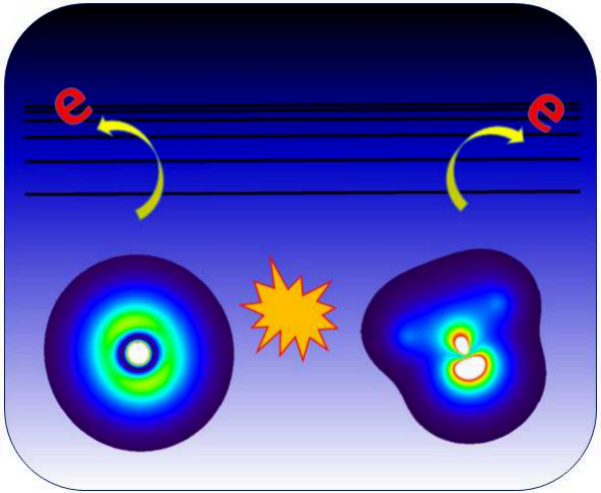

In this work, we investigate the capability of using real-time time-dependent density functional theory (RT-TDDFT) in conjunction with a complex absorbing potential (CAP) to simulate the intermolecular Coulombic decay (ICD) processes following the ionization of an inner-valence electron. We examine the ICD dynamics in a series of noncovalent bonded dimer systems, including hydrogen-bonded and purely van der Waals (VdW)-bonded systems. In comparison to previous work, we show that RT-TDDFT simulations with a CAP correctly capture the ICD phenomenon in systems exhibiting a stronger binding energy. The calculated time scales for ICD of the studied systems are in the range of 5–50 fs, in agreement with previous studies. However, there is a breakdown in the accuracy of the methodology for the pure VdW-bonded systems. Overall, the presented RT-TDDFT/CAP methodology provides a powerful tool for differentiating between competing electronic relaxation pathways following inner-valence or core ionization without necessitating any *a priori* assumptions.

The ionization of an inner-valence or core electron can initiate competing electronic relaxation pathways that occur on an ultrafast time scale, such as Auger–Meitner, intermolecular Coulombic decay (ICD), and electron-transfer-mediated decay (ETMD) processes.^1−3^ These processes play an important role in surface-science fragmentation and biological systems;^4,5^ the initial electron dynamics governs the subsequent fragmentation product distribution due to the Coulomb explosion of charged species in close proximity. In general, the majority of theoretical investigation of such processes has relied on static electronic structure calculations involving analysis of the ionization spectrum,^6−8^ density of states,^9^ or broadening of the electronic states through non-Hermitian techniques.^10−12^ However, with the advent of attosecond spectroscopy, it is now possible to have experimentally time-resolved observation of ultrafast processes with subfemtosecond resolution.^13−15^ Therefore, there is a benefit to developing practical simulation methods that go beyond static techniques and can more directly report on such experiments. Real-time electronic structure methods, which directly solve for the time propagation of the electronic wave function, provide a powerful class of techniques for accomplishing such a goal.^16−42^ In the context of electronic relaxation dynamics, a few highly accurate real-time studies of the explicit electronic motion have been performed on small systems, such as using the wave packet propagation method to simulate ICD in the Ne–Ar system^43^ and the MCTDH method for Fermions to simulate ICD in model potentials of quantum dots.^44−47^ Additionally, a few studies have used real-time time-dependent density functional theory (RT-TDDFT) with various levels of success.^48,49^

In this work, we develop a practical simulation protocol to use RT-TDDFT to simulate the real-time ICD dynamics following an inner-valence ionization event. The ICD process involves the initial ionization of a low-lying electronic level. The hole left behind is filled from an electron in a higher-lying electronic level on the same molecule. The energy released from this relaxation process is transferred to a neighboring molecule to ionize that molecule.^3^ In comparison to previous work, we employ a complex absorbing potential (CAP) to account for the ionized secondary electron and a tuned long-range corrected functional with diffuse basis functions to accurately capture the dynamics.

Figure 1 presents the variety of different dimer systems we examine, including both hydrogen-bonded and pure van der Waals (VdW)-bonded systems. The nuclear geometry for each dimer was obtained from the global minimum at the CCSD(T) level of theory from the corresponding references: H_2_O–H_2_O,^50^ HF–HF,^51^ Ar–H_2_O,^52^ Ne–H_2_O,^52^ and Ne–Ar.^53^ Each dimer is asymmetric, due to the involvement of two different species or the asymmetric hydrogen bonding geometry in the case of H_2_O–H_2_O and HF–HF. Therefore, to designate the two molecules in H_2_O–H_2_O and HF–HF, we label one molecule as the p-donor and one as the p-acceptor corresponding to the hydrogen-bonding donor and acceptor, respectively, in the dimer. As we will discuss below, we examine the electron relaxation dynamics following the inner-valence ionization of each molecule in the dimers.

**Figure 1:**
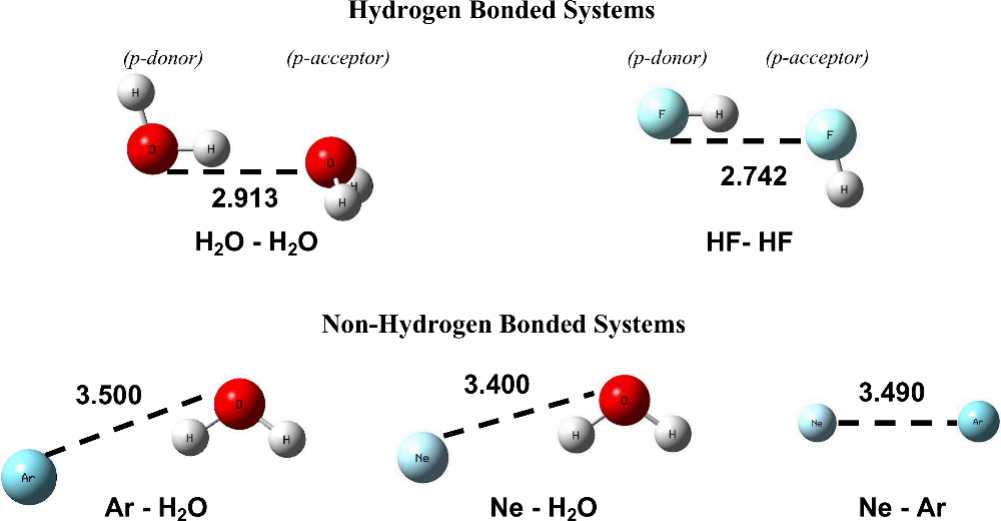
Geometry of the dimer systems investigated in this work, including hydrogen-bonded (top) and non-hydrogen-bonded (bottom) dimers. The heavy-atom internuclear distances are shown in angstroms. The monomers within the hydrogen-bonded dimers are designated as either the p-donor or the p-acceptor on the basis of the geometry.

We perform the following procedure to initialize the electronic system to account for the initial inner-valence ionization. First, we perform a ground-state density functional theory (DFT) calculation using the CCSD(T) geometries provided in Figure 1. These calculations, as well as the subsequent real-time calculations, are performed using LC-PBE*,^54^ where the asterisk indicates that we tune the range-separation parameter. Previous work has illustrated the importance of using a a long-range corrected functional, or at least some amount of exact exchange, to appropriately account for charge dynamics.^48,55,56^ We use the aug-cc-pVDZ basis^57^ for the molecule that will contain the initial hole (the trigger molecule), while the d-aug-cc-pVDZ basis^58^ is used for the molecule that will be ionized during the ICD process (the target molecule). However, given that the d-aug-cc-pVDZ basis does not exist for Ar, we instead use the def2-TZVPPD^52^ basis for the Ne–Ar system. Second, we generate the initial hole in the desired molecular orbital (MO) by manually changing the occupancy of that orbital in the one-electron-reduced density matrix (1RDM) obtained from the ground-state calculation; we find that the inner-valence MOs are well localized in all cases, making this choice relatively unambiguous. Full details of this procedure, including how we tune the range-separation parameter within LC-PBE*, and an investigation into our choice of functional and basis, are provided in the Supporting Information.

The real-time dynamics following the initial inner-valence ionization are simulated using RT-TDDFT as implemented in the NWChem^59^ simulation package. One of the main difficulties in performing this simulation is how to appropriately account for the ionization of the secondary electron in a finite basis. To overcome this difficulty, we use a CAP to effectively remove the electron from the system, as has been done to alleviate some of the numerical artifacts observed when simulating spectroscopy using RT-TDDFT.^42,55^ The modified RT-TDDFT equations of motion are given as
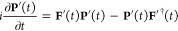
1where **P**′(*t*) is the 1RDM and **F**′(*t*) is a modified non-Hermitian DFT Fock matrix
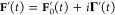
2The prime notation indicates that the matrices are defined in the basis of orthogonalized atomic orbitals (AOs); the relation to density matrix **P** and Fock matrix **F** in the standard AO basis is given through the procedure of canonical orthogonalization.^60,61^

Damping matrix **Γ**′(*t*) is obtained from a time-independent diagonal damping matrix **D**(42,55)
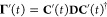
3where **C**′(*t*) corresponds to the eigenvectors of the time-dependent Fock matrix in the orthogonalized AO basis, **F**′(*t*). Diagonal matrix **D** contains exponentially increasing damping parameters (γ_*i*_), such that
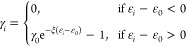
4To obtain the specific values for the terms γ_0_, ζ, and ε_0_ used to define the strength of the damping matrix, we follow an analogous protocol as employed in the context of spectroscopic calculations.^42,55^ Full details for the choice of these parameters along with the final values are provided in the Supporting Information.

We analyze the real-time dynamics in terms of (i) the occupancy of the MOs and (ii) the charge loss on each monomer. One issue in the former is that ICD is normally described in terms of a local molecular orbital picture. However, the valence molecular orbitals tend to be delocalized across both monomers. Therefore, to analyze the electron relaxation dynamics, we rotate the time-dependent 1RDM into the basis of ground-state MOs of each isolated monomer, such that
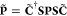
5Matrix **S** is the overlap matrix of the AOs, and **C̃** contains the combined MOs of each isolated monomer after an orthogonalization procedure as performed through NWChem’s “NOSCF” routine.

The charge loss on each monomer is defined as
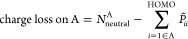
6where the sum runs over the MOs associated with monomer A. The sum is truncated at the HOMO associated with the original ground state of each monomer, and *N*_neutral_^A^ corresponds to the total number of electrons on neutral monomer A. This choice of truncation has a minimal impact on the observed results and is employed to minimize features arising with the choice of the CAP parameters.

We now turn our attention to the results generated using the RT-TDDFT workflow described above. Figure 2 presents the real-time dynamics for the H_2_O dimer following the initial ionization from the 2a_1_ MO on the p-donor (designated as water 1), which triggers a secondary ionization on the p-acceptor (designated as water 2). Figure 2a presents the total charge loss (black), along with the charge loss on water 1 (blue) and water 2 (red) (eq 6). The figure illustrates that initially, water 1 is in a cationic state while water 2 is neutral. Water 2 then begins to lose charge at the same rate as the total system loses charge, while water 1 remains in the cationic state. This is consistent with an ICD mechanism, indicating that our RT-TDDFT methodology can successfully capture the ICD process. The total charge-loss process occurs on a time scale of 20 fs, following which the charge on water 1 and water 2 begins to oscillate back and forth. The time scale is in agreement with previous estimates of the ICD process in the literature.^2^ The total charge loss saturates at a value of ∼1.6, which is below the expected final value of 2. However, given both the approximate nature of the dynamics and the presence of the CAP, a perfect final value of 2 is not expected and we consider the observed value of 1.6 a success.

**Figure 2:**
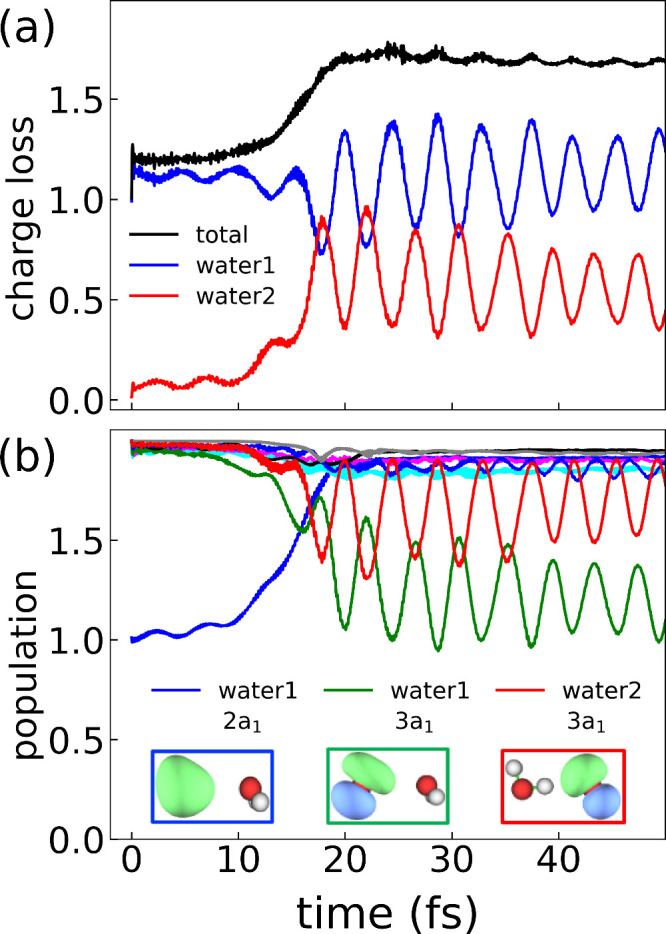
(a) Time-dependent charge loss (eq 6) on the water 1 (blue) and water 2 (red) monomers, which correspond to the p-donor and p-acceptor, respectively. The total charge loss of the H_2_O–H_2_O dimer (black) corresponds to the sum of the blue and red curves. (b) Time-dependent population of the MOs in the isolated monomer basis (eq 5) (water 1 2a_1_, blue; water 1 1b_1_, cyan; water 1 1b_2_, magenta; water 1 3a_1_, green; water 2 2a_1_, black; water 2 1b_1_, orange; water 2 1b_2_, gray; water 2 3a_1_, red). The insets show images of the MOs that are the dominant participants in the observed ICD mechanism. The orbitals are plotted using Multiwfn.^62^

We can further examine the mechanism of the electronic decay process by analyzing the RT-TDDFT trajectories in more detail. Figure 2b presents the time dependence of the MO occupation numbers in the isolated monomer basis, . An initial hole is generated in the 2a_1_ MO on water 1 (blue), which is repopulated by an electron relaxing from the valence 3a_1_ MO on water 1 (green). Simultaneously, the water 2 valence 3a_1_ (red) MO loses electron density associated with ionization out of the MO. We attribute the faster relaxation of an electron from the 3a_1_ MO, in comparison to relaxation from either the 1b_1_ or 1b_2_ MOs, to the matching orbital symmetry with the initial hole located on the 2a_1_ MO; this is in agreement with previous experimental results that revealed enhanced hole hopping in the 3a_1_ and 2a_1_ levels.^63^ This physical picture observed by the real-time dynamics is completely consistent with the expected ICD mechanism in the water dimer. Furthermore, the time scale of this whole process occurs on the order of 20 fs, in agreement with Figure 2a. Following the initial ICD process, we observe that the charge transfer pictured in panel a is mainly associated with charge transfer between the water 1 3a_1_ and water 2 3a_1_ MOs.

We illustrate one of the main benefits of a real-time investigation of electron relaxation dynamics by also discussing the results on the Ar–H_2_O system in detail (Figure 3). In this system, the initial hole is generated on the Ar 3s orbital and the water molecule is ionized during the ICD process. Figure 3a plots the charge loss on Ar (blue) and water (red). The general behavior, in which the Ar molecule remains effectively in a single cationic state while the water molecule loses charge, is consistent with an ICD process and what was observed in the H_2_O dimer (Figure 2). However, the time scale of ICD in the Ar–H_2_O system is slower, on the order of 40 fs; this result intuitively makes sense as the interaction between the non-hydrogen-bonded Ar–H_2_O system is weaker than in the H_2_O dimer. Interestingly, in comparison to the H_2_O dimer, Figure 2a also indicates additional dynamics on the time scale of the ICD process; the charge losses on Ar and water oscillate out of phase with each other, which is associated with charge transfer between the two species. The observed complicated dynamics and presence of ICD in the Ar–H_2_O system are consistent with previous experimental work examining water clusters adsorbed on rare-gas surfaces.^4^

**Figure 3:**
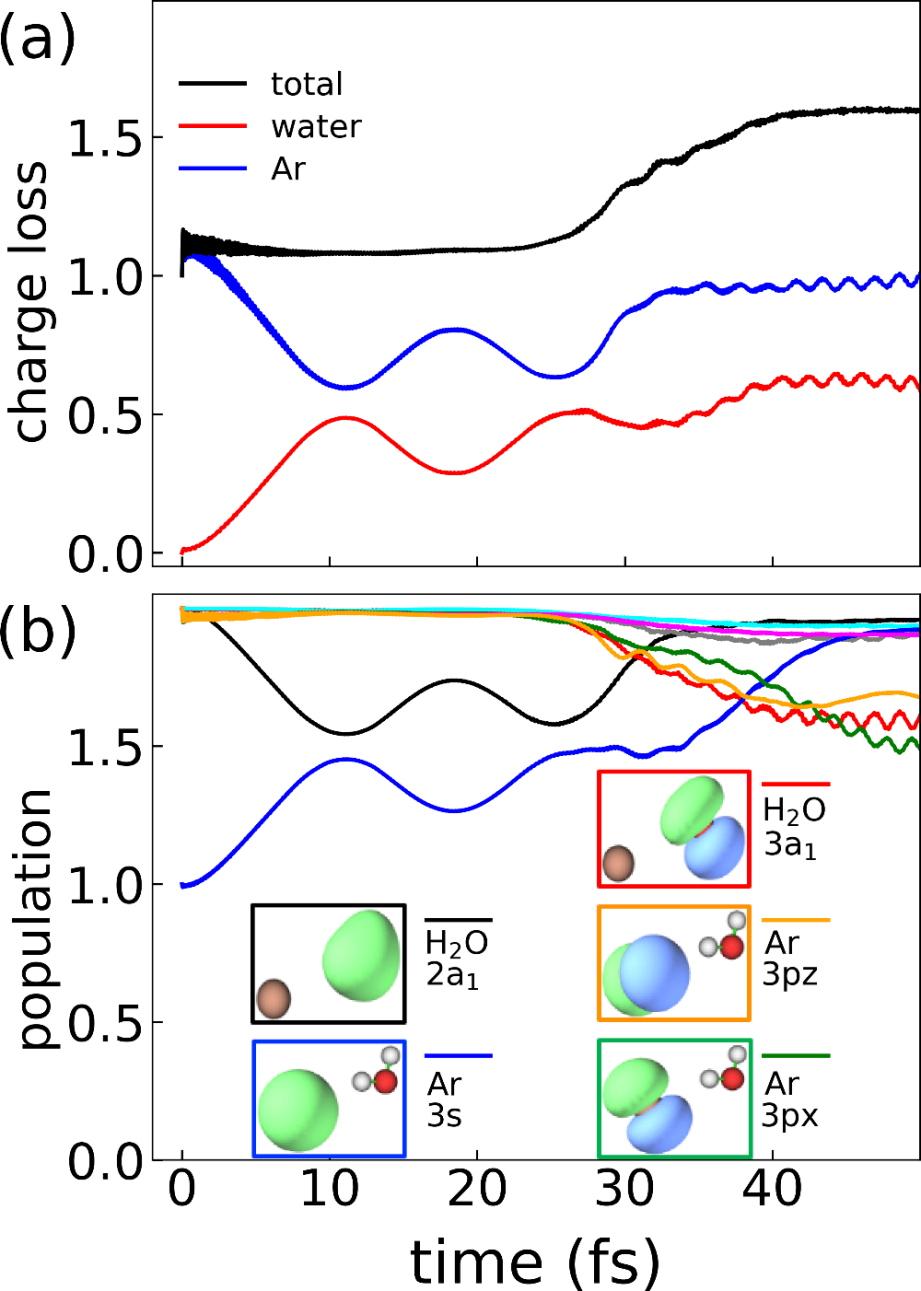
(a) Time-dependent charge loss (eq 6) on Ar (blue) and water (red). The total charge loss of the Ar–H_2_O dimer (black) corresponds to the sum of the blue and red curves. (b) Time-dependent population of the MOs in the isolated monomer basis (eq 5) (H_2_O 2a_1_, black; H_2_O 1b_1_, cyan; H_2_O 1b_2_, gray; H_2_O 3a_1_, red; Ar 3s, blue; Ar 3p_*x*_, green; Ar 3p_*y*_, magenta; Ar 3p_*z*_, orange). The insets show images of the MOs that are the dominant participants in the electron relaxation dynamics. The data show competition between hole transfer between Ar and H_2_O and the expected ICD mechanism. The orbitals are plotted using Multiwfn.^62^

Further insight into the complex dynamics is provided in Figure 3b, which presents the time-dependent MO populations. The initial hole on the Ar 3s orbital (blue) first exhibits Rabi oscillations associated with hole transfer to the water 2a_1_ MO (black), consistent with the charge transfer observed in Figure 3a. The Ar 3s orbital is then repopulated from electron density relaxing from the valence Ar 3p_*x*_ (orange) and 3p_*z*_ (green) orbitals, simultaneous with ionization of the valence water 3a_1_ MO (red). In this context, we observe hole transfer competing with the expected ICD process. The discovery and investigation of such competing pathways without any *a priori* knowledge of which pathways are important illustrate the major benefit of real-time methodologies, especially in more complex systems.

To illustrate that the observed hole-transfer dynamics is not simply an artifact of performing the analysis in the localized monomer basis, Figure 4 presents the time-dependent real-space hole density, which is insensitive to the choice of single-particle basis used to calculate the observable. We define the time-dependent real-space hole density as
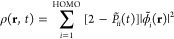
7where  is the occupation number of the *i*th orbital at time *t* and  is the wave function of the *i*th orbital. In the context of eq 7 and in Figure 4, we specifically perform the sum in the basis of the MOs of the isolated monomers (eq 5); the sum runs up to the HOMO associated with the initial ground-state calculation. However, an analogous plot in a delocalized MO basis provided results indistinguishable from those depicted in Figure 4.

**Figure 4:**
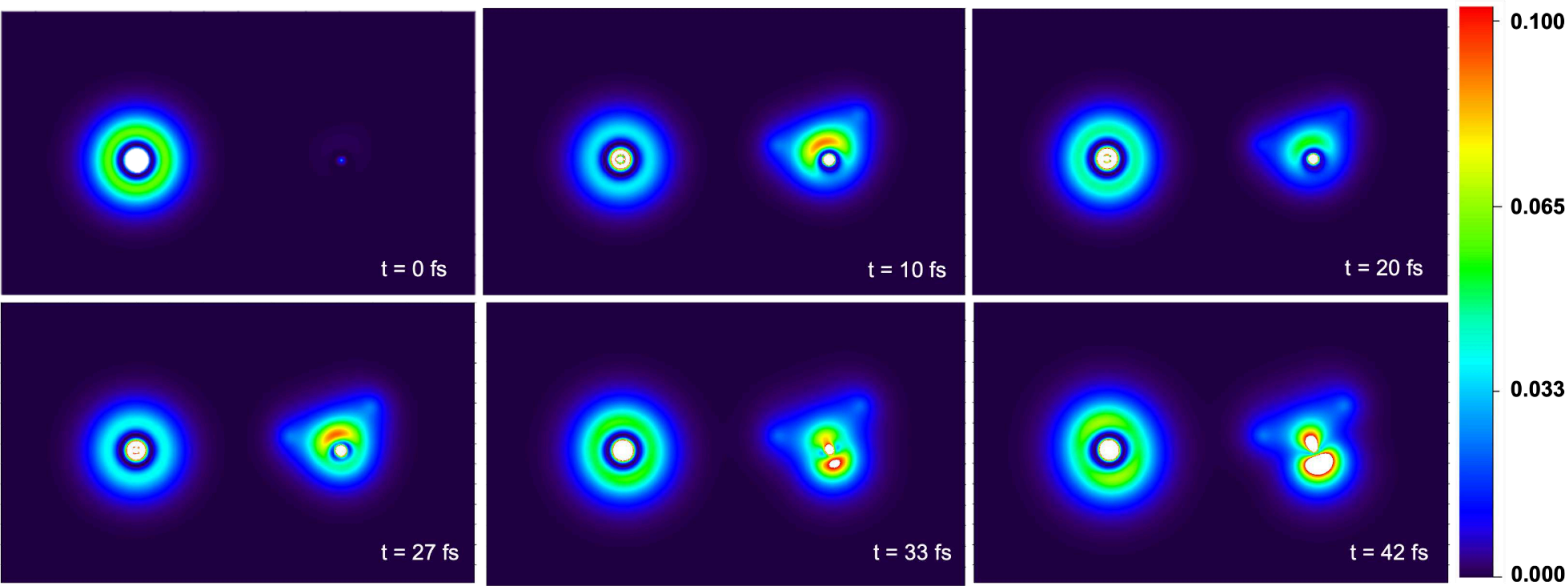
Heat map of the time-dependent real-space hole density (eq 7) of the Ar–H_2_O dimer. Specifically, the plot corresponds to a two-dimensional slice of the hole density corresponding to the plane of the Ar–H_2_O dimer. The frames depict different times along the RT-TDDFT trajectory illustrating that there is oscillatory hole transfer between Ar and H_2_O s-like orbitals (*t* = 0, 10, 20, and 27 fs), followed by an ICD mechanism to yield p-like hole densities on both Ar and water (*t* = 33 and 42 fs). The hole density snapshots are plotted using Multiwfn.^62^

As shown in Figure 4, the hole is initially located on Ar, exhibiting a spherical density pattern consistent with the 3s orbital. After 10 fs, the hole density partially transfers to the water molecule, exhibiting a distribution consistent with a localized 3a_1_ orbital. The charge oscillation can then be observed in the snapshots at 20 and 27 fs. Following the charge oscillation, the hole densities on Ar and water change to distributions consistent with more p-like orbitals at 33 fs and finally at 42 fs, in agreement with the ICD process pictured in Figure 3. The knowledge of the real-space density in comparison to the intermolecular distances may also provide a useful analysis tool as these parameters can have a strong influence on both the strength of the ICD coupling and the final Coulomb repulsion between species.^4^

Lastly, we examine the applicability of the developed RT-TDDFT methodology to correctly capture ICD dynamics in general systems. Figure 5 plots the time-dependent charge loss of the hydrogen-bonded H_2_O–H_2_O (left) and HF–HF (right) dimers. The top row corresponds to generation of the initial hole on the p-donor (blue), while the bottom row corresponds to generation of the initial hole on the p-acceptor (red). In general, we observe that the RT-TDDFT methodology correctly captures the ICD mechanism regardless of which monomer is initially ionized; the monomer with the initial hole stays in a cationic state, while the initial neutral monomer loses charge density associated with the secondary ionization process.

**Figure 5:**
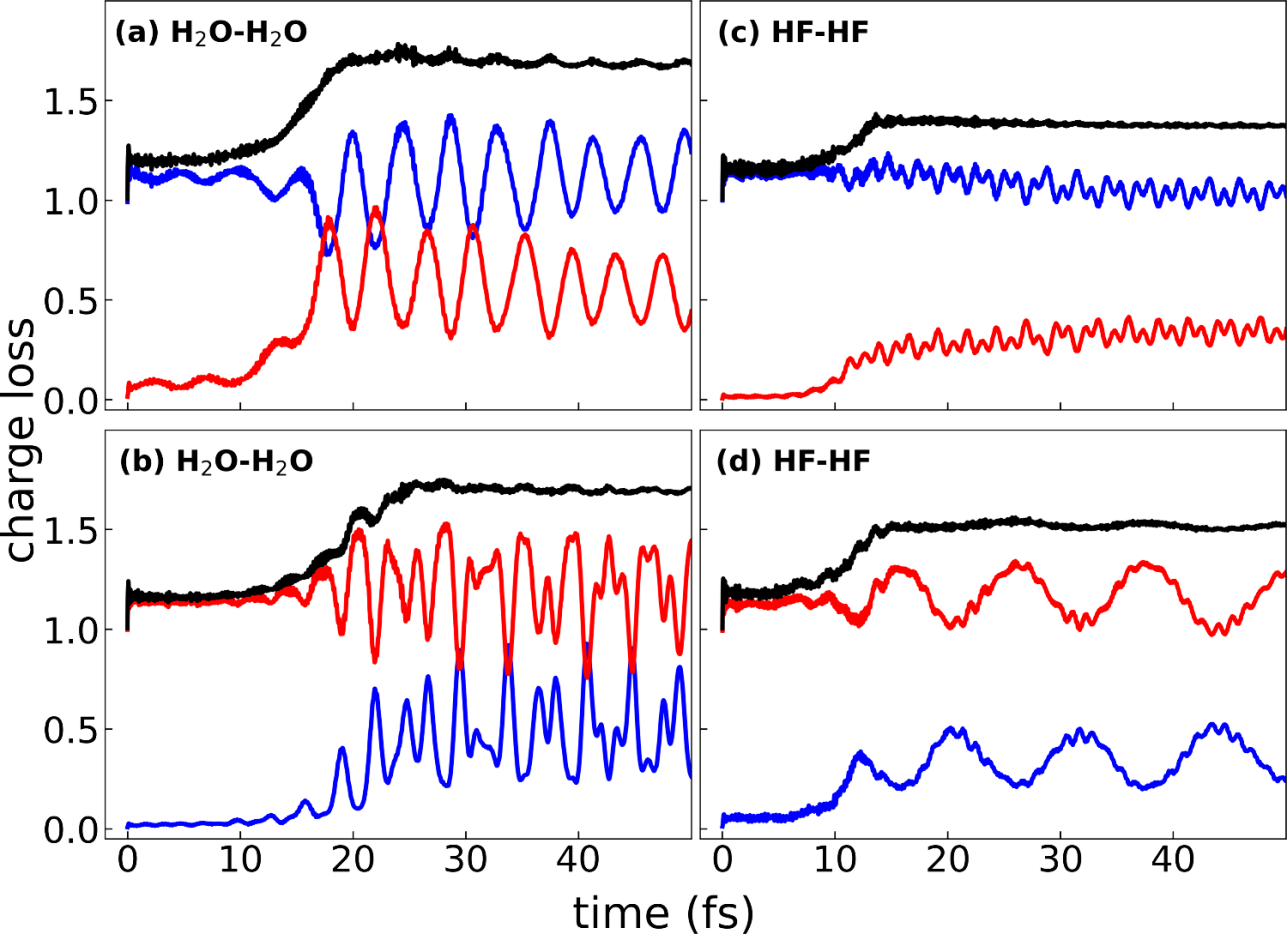
Time-dependent charge loss (eq 6) for the H_2_O–H_2_O (left column) and HF–HF (right column) dimers. The top row corresponds to generation of the initial hole on the p-donor (blue), while the bottom row corresponds to generation of the initial hole on the p-acceptor (red). The total charge loss of the dimer system (black) corresponds to the sum of the blue and red curves.

More specifically, both H_2_O–H_2_O cases yield an ICD process that occurs on a time scale of 20–30 fs; this is consistent with previous kinetic energy release (KER) experiments that show roughly equal kinetic energy of the ionized secondary electron in both cases.^2^ Similarly for the HF–HF dimer, both cases also show a clear ICD mechanism that occurs slightly faster, on the order of 15–20 fs, than in the case of the H_2_O–H_2_O dimer. This behavior is consistent with results for the HF trimer that indicate that ICD is possible both from a p-donor to a p-acceptor and vice versa.^7^

Figure 6 plots the time-dependent charge loss for two more weakly bound systems: (a) Ne–H_2_O and (b) Ne–Ar. In both cases, the initial hole is generated in the Ne 2s orbital (blue) and the charge loss of the other species is colored red. The presence of ICD in Ne–Ar has been well established^3,43,64−67^ and is also expected to occur in Ne–H_2_O.^68^ However, Figure 6 shows a breakdown of the developed RT-TDDFT methodology in correctly predicting the ICD mechanism in these systems. For Ne–H_2_O (Figure 6a), there is no energy exchange or secondary ionization observed. For Ne–Ar (Figure 6b), there is similarly no energy exchange, and instead, the Ne atom autoionizes, which should not be energetically feasible in this system. We attribute this breakdown to the weaker intermolecular forces in these systems, compared to those in the hydrogen-bonded systems, that are more challenging for DFT to correctly capture.^48^ This could be associated with an inaccurate treatment of the VdW interactions, which dominates the intermolecular interactions in these systems, or lack of an explicit treatment of electron correlation beyond what is captured by the exchange-correlation functional. Future development will attempt to alleviate these issues by exploring functionals that are explicitly developed for VdW interactions as well as the inclusion of screened Coulomb terms analogous to what has been done in previous applications of Auger–Meitner dynamics.^49^

**Figure 6:**
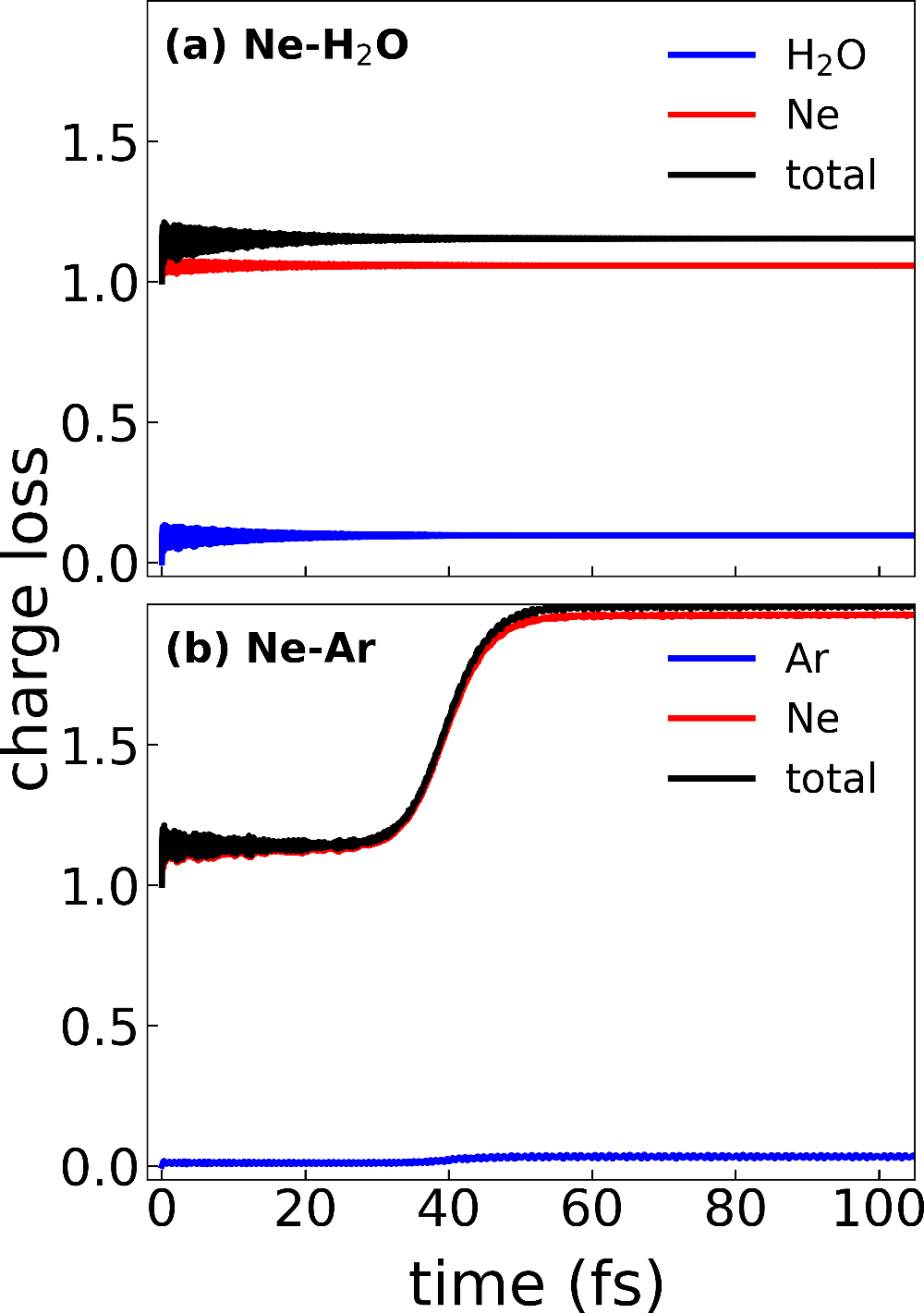
Time-dependent charge loss (eq 6) for (a) the Ne–H_2_O dimer and (b) the Ne–Ar dimer. In both cases, the initial hole is generated on Ne (blue) and the charge loss of the other species is colored red. The total charge loss of the dimer system (black) corresponds to the sum of the blue and red curves.

In summary, we introduce a RT-TDDFT methodology to directly simulate the ultrafast electron relaxation dynamics following an inner-valence ionization in real time. The methodology involves utilizing (i) a complex absorbing potential (CAP) to remove the secondary ionized electron, (ii) a long-range corrected functional with diffuse basis functions, and (iii) a localized MO basis to perform the analysis. We examine a series of noncovalently bonded dimer systems, including HF–HF, H_2_O–H_2_O, Ar–H_2_O, Ne–H_2_O, and Ne–Ar, and show that our methodology can correctly capture the ICD mechanism in the hydrogen-bonded and Ar–H_2_O systems, while the accuracy of the methodology breaks down in the more weakly bound Ne–H_2_O and Ne–Ar systems. As expected from previous work, we observe that the ICD process is dominant in all of the simulated dimer systems, such that no ETMD occurs. The presented methodology provides a useful tool for investigating competing electron relaxation pathways that is sufficiently efficient to examine complex systems.

## Supporting Information Available

Details of the choice of functional, range-separation parameters, and basis; additional results comparing different bases; and details of the choice and the values for the parameters of the complex absorbing potential (PDF)
