# Supplementary material for: Modeling Intermolecular Coulombic Decay with Non-Hermitian Real-Time Time-Dependent Density Functional Theory
Source: J Phys Chem Lett. 2024 Jul 25;15(30):7806–13. doi: 10.1021/acs.jpclett.4c01146 (PMC11299181; doi:10.1021/acs.jpclett.4c01146)
Supplement: Supplementary file 1 — jz4c01146_si_001.pdf [file jz4c01146_si_001.pdf]

Supporting Information: Modeling Intermolecular Coulombic Decay With Non-Hermitian Real-Time Time-Dependent Density Functional Theory

Yi-Siang Wang, James X. Zhong Manis, Matthew C. Rohan, Thomas M. Orlando, and Joshua S. Kretchmer*

School of Chemistry and Biochemistry, Georgia Institute of Technology, Atlanta, GA 30332, USA

E-mail: jkretchmer@gatech.edu

Initialization of the hole

We use the following procedure to generate the 1-electron reduced density matrix (1RDM), $\mathbf{P}$, that accounts for the initial ionization event: *(i)* We manually form a diagonal 1RDM, $\mathbf{P}_{\mathrm{MO}}$, with the diagonal elements given by either 2 or 0 corresponding to the occupied or virtual molecular orbital (MO)s. However, we change the diagonal element for the MO that corresponds to the ionized MO to 1 instead of 2. The MO indices correspond to those obtained from the ground-state DFT calculation on the dimer. *(ii)* This diagonal 1RDM is then rotated into the AO basis using

$$\mathbf{P} = \mathbf{C}\mathbf{P}_{\mathrm{MO}}\mathbf{C}^{\dagger}, \tag{1}$$

where **C** corresponds to the MO coefficient matrix in the atomic orbital (AO) basis obtained from the ground-state DFT calculation on the dimer. *(iii)* This final 1RDM, **P**, is then used as the starting point for the subsequent real-time time-dependent density functional theory (RT-TDDFT) calculation.

Choice of range-separation parameter

In this work we use the tuned long-range corrected LC-PBE* functional. We use a previously developed procedure to obtain a good choice of the range-separation parameter μ by minimizing the difference between the Koopmans' ionization potential and the true ionization potential.[1,2] This is done by minimizing the following object function

$$J(\mu) = |IP_{\mathrm{SCF}}(\mu) - IP_{\mathrm{Koopman}}(\mu)| = |E_{SCF}^{\mathrm{cation}}(\mu) - E_{\mathrm{SCF}}^{\mathrm{neutral}}(\mu) + \varepsilon_{\mathrm{HOMO}}^{\mathrm{neutral}}(\mu)|, \quad (2)$$

where the subscript SCF corresponds to a ground-state DFT energy and $\varepsilon_{\mathrm{HOMO}}^{\mathrm{neutral}}(\mu)$ is the energy of the HOMO from the neutral ground-state DFT calculation.

This procedure is performed for the various molecules studied in the manuscript. Specifically, the range-separation parameter is chosen for each molecule based on isolated monomer calculations. For the dimer systems, the range-separation parameter is chosen to be that of the monomer value associated with the target molecule. The final values of the range-separation parameter is provided in Table S1.

Table S1: The values of the range-separation parameter (μ), and the CAP parameters ε_0 and ξ. The molecule refers to the target molecule in each dimer.

Molecule	μ	ε_0	ζ
p-donor H_2O	0.51	0.047	0.5
p-acceptor H_2O	0.51	0.047	0.5
p-donor HF	0.64	0.024	0.1
p-acceptor HF	0.64	0.024	0.2
Ar	0.57	0.079	0.5

Choice of parameters in the complex absorbing potential

The diagonal damping matrix, D, used in the complex absorbing potential (CAP) has elements given by

$$\gamma_i = \begin{cases} 0, & \text{if } \varepsilon_i - \varepsilon_0 < 0 \\ \gamma_0 e^{-\xi(\varepsilon_i - \varepsilon_0)} - 1, & \text{if } \varepsilon_i - \varepsilon_0 > 0 \end{cases} \tag{3}$$

which corresponds to Eq. (4) in the main text. To obtain the specific values for the terms γ_0, ζ, and ε_0 used to define the strength of the damping matrix, we follow an analogous protocol as employed in the context of spectroscopic calculations.[1,3]

First, the value of γ_0 is always chosen to be 1 Ha, and the value of γ_i is capped to a maximum value of 100 Ha as was done previously.[3] We have observed that the choice of γ_0 has negligible influence on the dynamics even if the value is increased by an order of magnitude.

The value of the cut-off energy, ε_0, is chosen to partition the virtual orbitals into a set of bound or continuum orbitals based on their energy in comparison the following estimate of the electron affinity:[3]

$$EA_1 = E^{anion} - E^{neutral} \tag{4}$$

$$EA_k \simeq EA_1 + \nu_{k-1} \qquad k = 2, 3, \ldots \tag{5}$$

where ν_k corresponds to the kth TDDFT excitation of the anion. For a given value of k, EA_k and EA_{k+1} will switch from negative to positive. The value of ε_0 is chosen to match the interpolated value between ν_k and ν_{k+1}, such that the electron affinity equals zero. This procedure is performed on an isolated monomer and, analogous to the range-separation parameter, the value for a dimer system is chosen to be that of the monomer value associated with the target molecule. This procedure provides a physically motivated and unambiguous choice for the value of ε_0. The numerical results are insensitive to small changes in this choice. The values for each monomer are provided in Table S1.

Previous work focusing on spectroscopic calculations chose the value of the exponential decay parameter, ξ, large enough to remove spurious peaks in the spectra, but not so high to begin to alter the gross features. We take a similar tactic in the context of the calculations presented in this work. Specifically, we choose ξ to be as large as possible, but not so large to introduced artificial over-ionization of the system. A signature of artificial ionization is associated with the ionization of the trigger molecule from arbitrary MOs, which should not be an energetically feasible process in any of the systems studied herein. This is in comparison to the clean change in MO occupation associated with the intermolecular Coulombic decay (ICD) process presented in the main text. The final values of ξ are presented in Table 1.

Functional/basis set dependence

To choose a proper functional/basis set combination for capturing the ICD dynamics, we tested the following four combinations: LC-PBE*/aDZ-aDZ, LC-PBE*/aDZ-daDZ, LC-PBE*/daDZ-daDZ, and CAM-B3LYP/daDZ-daDZ on the water dimer with the p-acceptor acting as the trigger molecule. Here, aDZ and d-aDZ refer to the aug-cc-pVDZ and d-aug-cc-pVDZ basis sets, while the notation basis1-basis2 corresponds to using basis1 for the trigger molecule and basis2 for the target molecule. Figure S1 plots the charge loss on both water molecules along with the total charge loss for all four combinations. We observe analogous behavior in all four plots in terms of both the time-scale of the ICD process along with the final plateau values of the charge-loss on both water molecules. The exact nature of the high-frequency oscillations after $\sim$ 20 fs differ slightly between the different choices. However, given that these oscillations are most likely not physically relevant, we use LC-PBE*/aDZ-daDZ for all systems in our study to match previous work.[4]

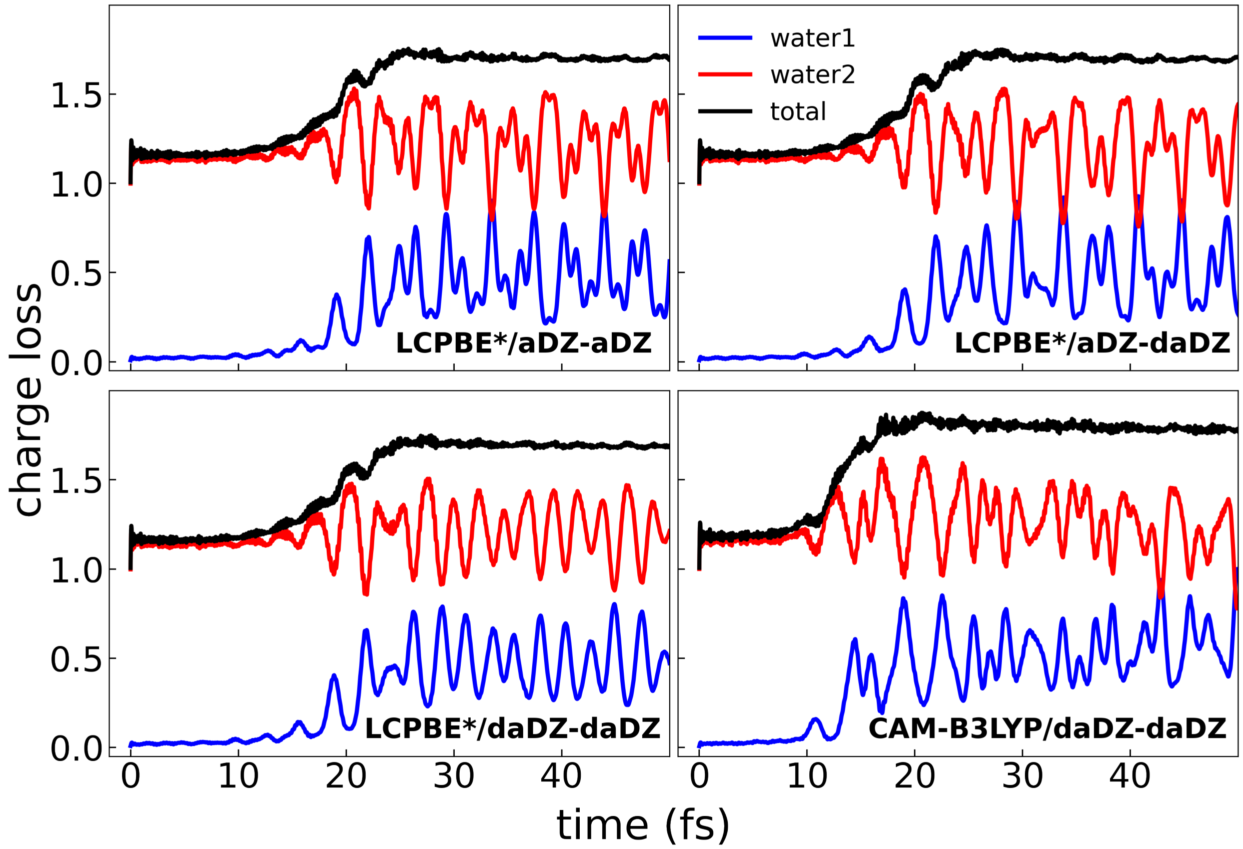

Figure S1: The time-dependent charge loss for the water dimer using different functional/basis set combinations.

References

(1) Lopata, K.; Govind, N. Near and Above Ionization Electronic Excitations with Non-Hermitian Real-Time Time-Dependent Density Functional Theory. *J. Chem. Theory Comput.* **2013**, *9*, 4939–4946.

(2) Lopata, K.; Govind, N. Modeling Fast Electron Dynamics with Real-Time Time-Dependent Density Functional Theory: Application to Small Molecules and Chromophores. *J. Chem. Theory Comput.* **2011**, *7*, 1344–1355.

(3) Fernando, R. G.; Balhoff, M. C.; Lopata, K. X-ray Absorption in Insulators with Non-Hermitian Real-Time Time-Dependent Density Functional Theory. *J. Chem. Theory Comput.* **2015**, *11*, 646–654.

(4) Kuleff, A. I.; Cederbaum, L. S. Tracing Ultrafast Interatomic Electronic Decay Processes in Real Time and Space. *Phys. Rev. Lett.* **2007**, *98*, 083201.
